# Boolean logic in synthetic biology and biomaterials: Towards living materials in mammalian cell therapeutics

**DOI:** 10.1002/ctm2.1244

**Published:** 2023-06-29

**Authors:** Eric M. Bressler, Sarah Adams, Rong Liu, Yolonda L. Colson, Wilson W. Wong, Mark W. Grinstaff

**Affiliations:** ^1^ Department of Biomedical Engineering and Biological Design Center Boston University Boston Massachusetts USA; ^2^ Division of Thoracic Surgery Department of Surgery Massachusetts General Hospital Harvard Medical School Boston Massachusetts USA; ^3^ Department of Chemistry and Department of Biomedical Engineering Boston University Boston Massachusetts USA

**Keywords:** biomaterials, boolean logic, CAR T cells, cell therapy, drug delivery, synthetic biology

## Abstract

**Background**: The intersection of synthetic biology and biomaterials promises to enhance safety and efficacy in novel therapeutics. Both fields increasingly employ Boolean logic, which allows for specific therapeutic outputs (e.g., drug release, peptide synthesis) in response to inputs such as disease markers or bio‐orthogonal stimuli. Examples include stimuli‐responsive drug delivery devices and logic‐gated chimeric antigen receptor (CAR) T cells. In this review, we explore recent manuscripts highlighting the potential of synthetic biology and biomaterials with Boolean logic to create novel and efficacious living therapeutics.

**Main body**: Collaborations in synthetic biology and biomaterials have led to significant advancements in drug delivery and cell therapy. Borrowing from synthetic biology, researchers have created Boolean‐responsive biomaterials sensitive to multiple inputs including pH, light, enzymes and more to produce functional outputs such as degradation, gel‐sol transition and conformational change. Biomaterials also enhance synthetic biology, particularly CAR T and adoptive T cell therapy, by modulating therapeutic immune cells in vivo. Nanoparticles and hydrogels also enable in situ generation of CAR T cells, which promises to drive down production costs and expand access to these therapies to a larger population. Biomaterials are also used to interface with logic‐gated CAR T cell therapies, creating controllable cellular therapies that enhance safety and efficacy. Finally, designer cells acting as living therapeutic factories benefit from biomaterials that improve biocompatibility and stability in vivo.

**Conclusion**: By using Boolean logic in both cellular therapy and drug delivery devices, researchers have achieved better safety and efficacy outcomes. While early projects show incredible promise, coordination between these fields is ongoing and growing. We expect that these collaborations will continue to grow and realize the next generation of living biomaterial therapeutics.

## Graphical abstract

The intersection of synthetic biology and biomaterials promises to enhance safety and efficacy in novel therapeutics. By using Boolean logic in both cellular therapy and drug delivery devices, researchers have achieved better safety and efficacy outcomes. We expect that these collaborations will continue to grow and realize the next generation of living biomaterial therapeutics.

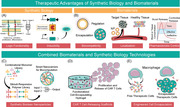

## 1 INTRODUCTION

### 1.1 The emerging collaboration of biomaterials and synthetic biology

**FIGURE 1:**
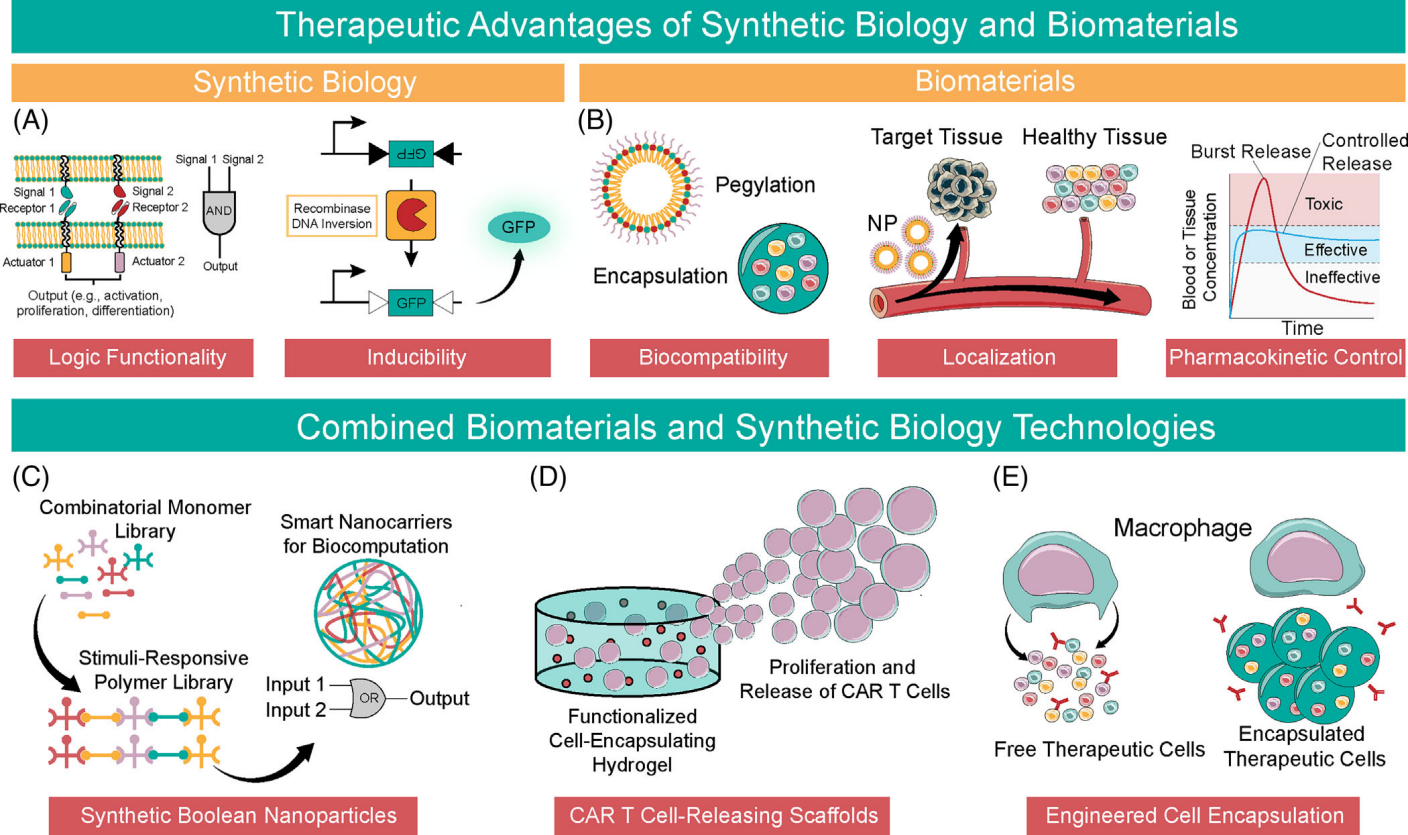
Collaborative advances in synthetic biology and biomaterials. (A) Synthetic biology confers control over therapeutic systems via Boolean logic and inducibility. Shown is a split chimeric antigen receptor (CAR) T cell that exhibits AND gate Boolean logic by splitting activation signals, requiring both for activation (figure adapted from Cho et al.) (left) and recombinase‐based inducible gene circuits (figure adapted from Weinberg et al.) (right) (B) Biomaterials offer biocompatibility via pegylation, encapsulation, biomimicry and rational design. Biomaterials also confer enhanced biodistribution and pharmacokinetics through targeted therapies and controlled release systems. (C–E) Existing technologies that harness strengths of both synthetic biology and biomaterials include (C) synthetic Boolean‐responsive nanoparticles (adapted from Zhang et al.), (D) CAR T cell‐releasing scaffolds (adapted from Grosskopf et al. and Agarwalla et al.), and (E) encapsulated designer cell therapies.

The fields of biomaterials and synthetic biology are rapidly converging on overlapping areas of translational medicine. The rise of stimuli‐responsive materials and cell‐based computation has forged collaborative pathways between these largely independent fields. Biomaterials are one of the oldest disciplines in biomedical engineering, with roots as far back as antiquity when Egyptian physicians used sutures made from animal sinew and prostheses made of wood.^1^, ^2^ Meanwhile, synthetic biology emerged in the early 2000s with the creation of a toggle switch^3^ and a repressilator^4^ in *Escherichia coli (E. coli)*. Despite the disparate origins of these fields, logic, inducibility, biocompatibility and ideal biodistribution and pharmacokinetics are universally desirable in therapeutic systems (Figure 1A,B).

Modern biomaterials emerged from an inherent need for biocompatibility, localization, and pharmacokinetic control, from implants such as intraocular lenses, joint prostheses and stents^5^ to polymers for controlled release.^6^, ^7^ Synthetic biology therapeutics promise substantial advantages over small molecule drugs and biologics. Early examples, free from the constraints of biocompatibility, pharmacokinetics and variable mechanical forces of the human body, demonstrate incredible displays of biocomputation and programmability, including Boolean logic at the gene and protein level and drug‐inducible systems.^8^, ^9^, ^10^, ^11^ Although recent developments in biomaterials confer stimuli‐responsive properties and Boolean logic, even cutting‐edge biomaterials lack the precision and programmability of genetic circuits. Thus, in recent years, the marriage of these two disciplines has harnessed the programmability of synthetic biology with the biocompatibility and pharmacokinetic control of biomaterials. Further, there is a clear convergence towards Boolean‐logic and biocomputation in both fields. Recent advances include Boolean responsive nanocarriers^12^ (Figure 1C), chimeric antigen receptor (CAR) T cell encapsulating hydrogels^13^, ^14^ (Figure 1D) and alginate‐encapsulated designer therapeutic cells^15^, ^16^ (Figure 1E). Boolean‐responsive biopolymers and biomaterial accessories for cellular therapy herald the synergistic potential of these fields.

Herein, we briefly review developments in each field, focusing on collaborations between synthetic biology and biomaterials. We place a special focus on biomaterials for drug delivery and synthetic biology for modulation of therapeutic T cells because of considerable collaboration and clinical development in these areas. Finally, we examine how these developments apply to new fields within cellular therapeutics and drug delivery, particularly the development of living materials. While there are many developments in bacterial biomaterial systems, the topic has been reviewed elsewhere, and we have limited the current discussion to mammalian systems.^17^, ^18^, ^19^

### 1.2 Smart and synthetic biomaterials for programmability in drug delivery

Biomaterials confer augmented control of pharmacokinetics, allowing clinicians and scientists to manipulate the ‘where’, ‘when’, and ‘how much’ of drug delivery. The current generation of drug delivery biomaterials includes polymer‐conjugated proteins,^20^ biomimetic polymers^21^, ^22^ and drug depots such as hydrogels,^23^, ^24^ particles^25^, ^26^, ^27^ or scaffolds.^28^, ^29^ While this approach has yielded many United States Food and Drug Administration (FDA)‐approved drugs, achieving greater precision and site‐specific delivery requires programmable materials that respond to environmental or biological cues or user input.

Programmable, ‘smart’ biomaterials have arisen in recent years. Biomaterials exhibit responsiveness and even Boolean algebra through environmental inputs.^30^ The need for ‘smart’ biomaterials arose from the natural limitations of traditional biomaterial systems. Once introduced to a biological system, traditional biomaterials interact with surrounding cells without opportunity for modulation or intervention, with potential adverse outcomes such as inflammatory response^31^ and release of cargo in off‐target tissues.^32^ Responsive biomaterials not only promise to ameliorate such limitations but also expand capabilities. These properties mimic many of the strengths of synthetic biology, demonstrating a clear need for programmability in biomaterials applications.

### 1.3 Cell‐based therapy enhanced by functional biomaterials

Synthetic biology champions programmability as its principal strength, affording control of synthetic gene circuits composed of layered Boolean logic gates via inputs such as small molecule drugs, biomarkers, light, and others. Though developed in bacteria, these systems are now optimized for mammalian cells, paving the way for synthetic biology within cellular therapy. The most prominent example is CAR T cells. CAR T cells express a synthetic receptor that activates natural T cell pathways upon binding a particular antigen. As of 2022, there are six FDA‐approved CAR T cell therapies for leukemia, lymphoma and multiple myeloma.^33^ Beyond cancer, both CAR and designer cells promise to expand cell therapy to endocrine and metabolic disorders, autoimmune diseases and infectious diseases.^15^

With few exceptions, cell‐based therapies need a substrate for activation, recognition or encapsulation to attenuate the appropriate response. While natural or autologous materials may augment cellular therapy, biomaterials represent a low‐cost, off‐the‐shelf alternative. A prominent example among FDA‐approved therapies is T cell activation via magnetic beads coated with activating signals, replacing an antigen‐presenting cell. Another example in development is semi‐permeable encapsulation materials for in situ cell‐based manufacture of biologically active peptides (e.g., insulin from pancreatic β cells).^34^, ^35^

Current examples of synergy between biomaterials and synthetic biology systems demonstrate the potential for augmentation of existing therapeutic systems. The following sections cover specific technologies and explore how the fields can leverage these technologies to achieve larger, overarching goals of biomaterials and synthetic biology in the therapeutic space.

## 2 BOOLEAN LOGIC IN BIOMATERIALS

### 2.1 Environmentally responsive biomaterials: From analog materials to YES gates

**FIGURE 2:**
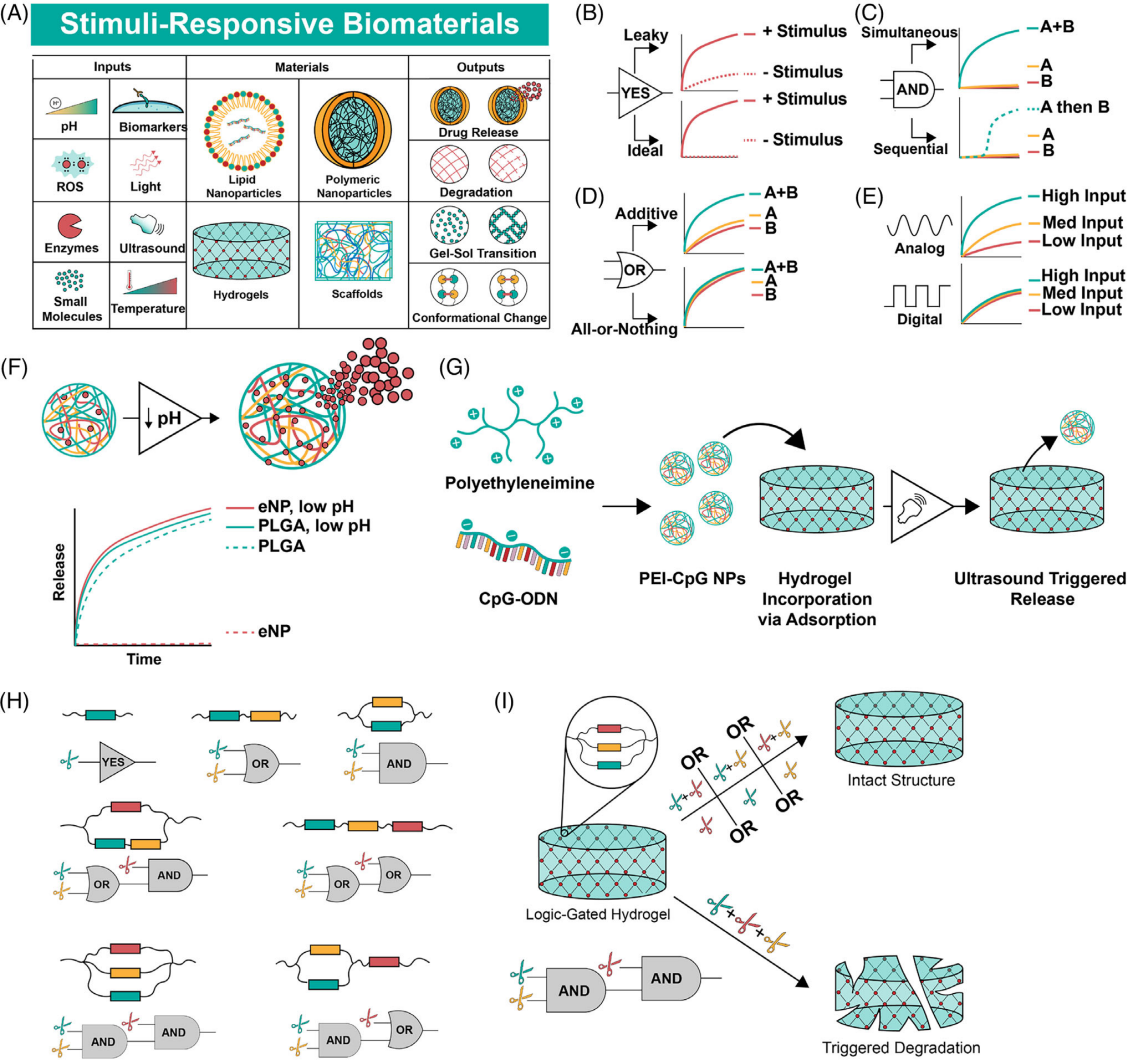
Building programmable, Boolean‐responsive biomaterials using stimuli‐responsive domains. (A) Stimuli responsive biomaterials utilize many inputs to achieve outputs via modulating the physical and chemical properties of the material. (B) YES gates in biomaterials often display leaky output. (C) AND gates are designed for simultaneous release, but many biomaterial AND gates utilize sequential release to match conditions of a physiologic scenario. (D) OR gates ideally exhibit all‐or‐nothing responses, but often result in additive responses instead, where presence of both signals leads to a larger effect. (E) Stimuli responsive materials usually respond in a concentration dependent manner, which emulates an analog signal. Digital signaling requires a single output strength regardless of input concentration. (F) An expansile nanoparticle releases cargo only when exposed to low pH. Adapted from Griset et al. (G) Polyethyleneimine‐CpG (PEI‐CpG) NPs loaded by adsorption into hydrogel/cryogel formulations, which are (H) synthetic polymers with cleavable domains create YES, OR, AND, and stacked logic gates. (I) Logic‐gated polymers from (G) are used as cleavable linkers in a hydrogel to enable logic‐gated degradation. (H and I) Adapted from Badeau et al. All data represented are idealized.

Imparting logic into materials is a natural extension of environmentally responsive materials that have been in development for decades (Figure 2A).^30^, ^36^ Some of the earliest examples of environmentally responsive materials are polymers that change conformation upon exposure to pH, ionic or temperature changes, such as poly(aminocarboxylic acid), (hydroxyethyl)methacrylate (HEMA) and methacrylic acid (MAA) copolymers and N‐alkyl acrylamide polymers, respectively.^37^ Countless physiologically responsive polymers have since been synthesized. Poly(lactic‐glycolic) acid (PLGA) micro‐ and nanoparticles are prototypical drug delivery devices that hydrolyze and release cargo at a faster rate at low pH.^38^ Prominent FDA‐approved PLGA‐based drug depots include Lupron depot, Eligard and Zoladex among others. While environmentally responsive materials are abundant, most exhibit leaky behavior (Figure 2B). For instance, a reactive oxygen species‐sensitive hydrogel may release faster in the presence of hydroxide, but the release is not completely inhibited in a physiologic buffer.^24^ OR‐gated materials may show an additive response instead of a consistent response regardless of inputs (Figure 2C), and AND‐gated materials often rely on sequential or ordered inputs (Figure 2D). Finally, programmable biomaterials typically exhibit dose‐dependent outputs, which mimic analog behavior. However, digital behavior requires responses that do not vary with input concentration (Figure 2E). Achieving programmability with biomaterials began with YES gates, or stimuli responsive biomaterials that exhibit explicit ON‐OFF behavior.

#### 2.1.1 ON‐OFF behavior via physiologic cues

Physiologic conditions such as pH and enzyme concentration are used to drive ON‐OFF behavior in materials. Local pH varies within physiologic environments. Thus, engineered materials respond to distinct environments based on local pH.^39^ Normal physiologic pH in blood and extracellular fluid maintains a tight distribution between 7.35–7.45. Meanwhile, the tumour microenvironment (TME) exhibits a pH range of 6.5–7.2, and lysosomes and endosomes measure between 5.0 and 6.5. Other environments such as the gastrointestinal tract, skin and vagina exhibit variable pH.

The earliest pH‐responsive materials rarely possessed the ON‐OFF characteristics necessary for building Boolean systems. These materials typically relied on varying breakdown kinetics via acid‐labile polymer backbones as targets for degradation.^40^ pH‐responsive materials that exhibit a stepwise response to environmental changes (YES gate) often do so through protonation/deprotonation to trigger a distinct, reversible change.^30^, ^41^ For instance, Angelos et al. describe mesoporous silica nanoparticles (MSNs) with nanovalves that opened below pH 5.4 but exhibit no release at pH 6.5.^42^ Gan et al. report MSNs with similar characteristics around pH 3, which switch between ON and OFF states by adjusting pH.^43^ In 2009, Griset et al. report expansile polymeric nanoparticles, which swell at pH 5 but exhibit a tight structure at physiologic pH, retaining their cargo. When loaded with paclitaxel, the drug releases in low pH buffer only and retains nearly all drug in neutral buffer, demonstrating clear ON‐OFF behavior.^44^ This system is efficacious in models of ovarian cancer,^45^ pancreatic cancer^46^ and mesothelioma^47^ (Figure 2F).

West and Hubbell first described specific enzyme‐degradable polymeric block co‐polymers in 1999, establishing a new class of stimuli‐responsive biomaterials.^48^ Like pH, enzymes exhibit heterogeneous distribution, enabling spatial and temporal control over cleavage. For instance, matrix metalloproteinases (MMPs), hyaluronidase, cathepsin B and esterase are overexpressed in tumour tissue.^49^ However, enzyme‐based responses can exhibit digital behavior as enzymes are more likely to be either present or absent, or active or repressed. Further, enzyme‐triggered materials are attractive due to mild reaction conditions and high substrate specificity.^50^

MMP‐labile peptides are used for cell‐mediated structural remodeling of extracellular matrix (ECM)‐like scaffolds and as a trigger for material degradation. Koshy et al. created an MMP‐degradable, cell‐responsive cryogel that releases granulocyte‐macrophage colony‐stimulating factor to draw immune cells into the porous matrix.^51^ Other enzymes, including hyaluronidase, cathepsin B and esterase, act as stimuli for the modulation of biomaterials. Li et al. report NPs made from hyaluronic acid (HA) coupled to the fluorophore chlorin e6 (HA‐Ce6 NPs). ‘ON/OFF’ degradation and fluorescence quenching and dequenching occur in the presence and absence of hyaluronidase in vitro and demonstrated anti‐tumour efficacy in a model of A549 lung cancer in vivo.^52^

#### 2.1.2 ON‐OFF behavior via bio‐orthogonal triggers

Bio‐orthogonal triggers (e.g., light, chemical inducers) confer direct control to the end‐user of a responsive material, potentially affording greater specificity and fewer off‐target effects. Photo‐sensitive biomaterials degrade or crosslink upon exposure to light, often near‐infrared light as it is low‐energy and penetrates tissue on the order of 1–2 centimeters.^53^ Polymer networks, particularly, hydrogels, are common light‐responsive biomaterials. Several researchers report light‐inducible stiffening,^54^ softening^55^ and reversible^56^ hydrogel networks, typically using o‐nitrobenzyl as a photodegradation tag and methacrylate or other functionalized molecules (thiol, dibenzocyclooctyne‐azide couples, or azobenzene) as a stiffening/crosslinking agent.

Ultrasound is another non‐invasive strategy for modulating biomaterial responses, chosen for its deep tissue penetration, millimeter‐scale spatial resolution, and high potential for clinical application.^57^ Ultrasound produces pressure waves, which trigger many responses in engineered materials including controlled release and gelation. For instance, cryogels loaded with cytosine‐phosphodiester‐guanine‐oligonucleotide (CpG‐ODN) complexes exhibit ultrasound‐triggered release as a cancer vaccine.^58^ Similarly, superhydrophobic meshes release small molecule drugs in response to high intensity focused ultrasound.^59^

### 2.2 Boolean biomaterial logic gates

Environmentally responsive biomaterials effectively form YES gates. However, to build Boolean logic systems, biomaterials must respond to multiple inputs to form AND, OR, NIMPLY and other logic gates. Further, the materials must respond independently and not subsequently or in coordination. As the structure of responsive elements is distinct for each trigger, multi‐input responsive biomaterials typically utilize different categories of triggers to induce orthogonal reactions.

#### 2.2.1 OR gates

An OR gate produces an output from sensing one of two inputs. While many multi‐stimuli‐responsive biomaterials systems exist, OR logic requires that either stimulus is sufficient to induce a response and that they induce the same response (e.g., disassembly of a particle, degradation of a hydrogel). There are many excellent reviews, which discuss multi‐stimuli‐responsive biomaterials, and the reader is referred to these articles.^60^, ^61^

OR‐gated biomaterials are a subset of multi‐stimuli‐responsive biomaterials. Guo et al. report dual responsive boronate‐phenolic acid capsules, which respond to either acidic pH or excess *cis*‐diols to induce disassembly of the capsule. The boronate‐phenolic ester network rapidly dissociates due to either low pH, which shifts the equilibrium between hydrophobic and hydrophilic properties of phenylboronic acid, or due to excess *cis‐*diols, which competes with *cis*‐diols incorporated into the capsule structure.^62^ In another example, dual‐sensitive micelles composed of disulfide‐bonded poly(β‐amino ester)s exhibit faster doxorubicin release at low pH (<6.5) or in the presence of dithiothreitol (DTT).^63^

While these materials form dual‐responsive systems that produce similar outputs from distinct inputs, compared to genetic circuits they still exhibit high levels of basal activity and analog responses based on the quantity of inducer present. For example, at 10 h, the boronate‐phenolic ester network capsules exhibit approximately 15% cargo release at pH 7.4 compared to approximately 60% at pH 5.0. Thus, a large pH change results in only a 4‐fold increase in release rate.^62^ Nearly all dual‐responsive drug delivery materials display similar characteristics, unlike genetic circuits, which rely on tightly regulated transcriptional units and regularly achieve log‐scale fold‐change between ON and OFF states. Further, many systems, which resemble OR logic, do not possess an equivalent response for each input or exhibit analog responses to each input, demonstrating that more specific strategies are necessary to form the building blocks for biocomputation with these systems.

#### 2.2.2 AND gates

AND gates require two inputs for a single output. Within biomaterials and synthetic biology, AND gates enhance specificity to increase efficacy and prevent off‐target toxicity. Strategies for creating AND‐gated biomaterials involve layered stimuli responses induced by pH, redox reactions, enzymes and light. Like stimuli‐responsive biomaterials, some of the earliest demonstrations of AND‐gated biomaterials were in MSNs. Dual‐controlled MSNs, synthesized by Angelos et al. use light‐responsive nanoimpellers and pH‐responsive nanovalves, which only release the contents of nanopores within the MSNs upon exposure to high pH AND light at 448 nm.^64^ While this early demonstration laid the foundation for further development of AND‐gated materials, alkaline pH is rare in human physiology, and green light shows poor depth of penetration in tissue. Several years later, Chen et al. reported core‐shell MSNs from polycaprolactone (core) polyacrylic acid (shell) as esterase‐ and pH‐sensitive layers, respectively. These particles release doxorubicin only in the presence of low pH and esterase, achieving AND logic under physiologic conditions. The particles demonstrate specific in vitro cytotoxicity against neuroblastoma cells compared to non‐cancerous MRC‐5 human fetal lung fibroblasts.^65^

AND logic is demonstrated in polymeric systems also. Wei et al. report a polymer micelle from a hydrazine functionalized poly(ethylene glycol)‐b‐poly(methyl methacrylate) (PEO‐b‐PMAA) copolymer attached to doxorubicin and crosslinked with dithiodiethanoic acid. DTT reduces the disulfide bonds and dissociates the particle structure, but under normal pH conditions the drug cargo remains covalently attached. The presence of low pH cleaves the drug from the polymer backbone, but intact particles retain the drug. Thus, the polymer micelle delivers doxorubicin in a pH‐ and reduction‐dependent manner, releasing most cargo only under low pH and high DTT conditions. However, the release of doxorubicin still occurs in a leaky manner in a 15 mM DTT solution.^66^

### 2.3 Complex biocomputation with supramolecular materials

Materials biocomputation, performing multi‐input logic functions with biomaterials, is in its infancy. However, there is an explosion of interest in polymer materials exhibiting logic computation and programmability.^30^, ^67^, ^68^ Such systems resemble early projects in synthetic biology, performing simple logic operations that are layered for complex computation. Unlike traditional stimuli‐responsive materials, these systems exhibit programmability, enabling diverse logic functionality with minimal changes to structure or inputs.

Early supramolecular logic gates were achieved via gel‐sol transitions based on distinct inputs. Komatsu et al. describe a hydrogel using a phosphate‐type hydrogelator, which undergoes a gel‐sol transition via temperature, pH, Ca^2+^ and light, demonstrating AND, OR, NAND and NOR logic responses.^69^ Ikeda et al. demonstrate OR and AND logic via a nanofiber network, which collapses upon exposure to hydroxide. The networks were made redox sensitive and loaded with enzymes such as oxidases (e.g., glucose oxidase, GOx and choline oxidase, COx) or reductases (e.g., nitroreductase, NR). This enabled collapse upon addition of a substrate (e.g., glucose, choline, nicotinamide adenine dinucleotide hydrogen (NADH)). The system exhibits OR‐gated collapse via GOx and COx encapsulation and AND‐gated collapse via mixing NR and GOx encapsulating gels.^70^

Programmable polymer libraries use cross‐linkers spatially configured to induce biomaterial degradation upon exposure to triggers, with the ability to exhibit YES, OR and AND logic and all possible two‐layered combinations of those gates (Figure 2G, Badeau et al.). These hydrogels use linear or cyclic cross‐linkers that promote hydrogel structure unless fully cleaved, presenting an opportunity for logic functions by placing inducible cleavage sites in series (OR‐logic) or in parallel (AND logic). The group uses linkers cleaved by enzymes (MMPs), ortho‐nitrobenzyl esters and ultraviolet light and demonstrates release of small molecule drugs and live cells with high spatial and temporal specificity (Figure 2H).^71^ Ruskowitz et al. utilize a similar hydrogel system to achieve modular release of tethered prodrugs via reduction reactions, enzymes or light,^72^ and Gawade et al. demonstrate protein release from a homogeneous hydrogel material using light, enzymatic and reductant triggers to achieve YES, OR and AND logic and spatially controlled and sequential release.^73^ Nanocarriers containing layered logic gates and sequential responsiveness using self‐immolative motifs, reported by Zhang et al., exhibit YES, OR, AND and multi‐layer logic and respond to pH, reductants, light and enzymes. Localized release of diverse cargo such as small molecules and RNA demonstrates anti‐tumour efficacy in vitro and in vivo.^12^

## 3 CELLULAR LOGIC AND SYNTHETIC BIOLOGY ENHANCED WITH BIOMATERIALS

**FIGURE 3:**
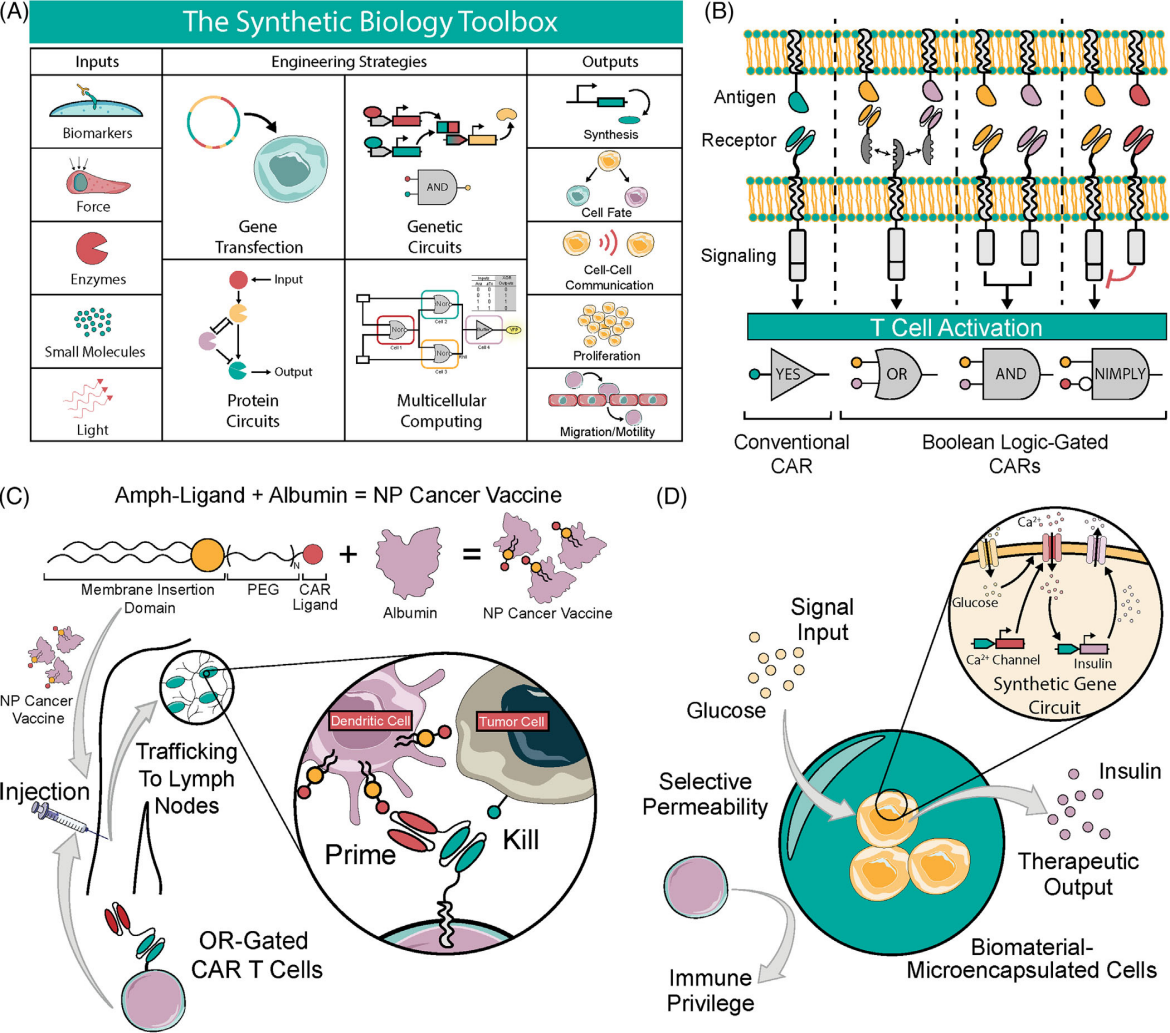
Synthetic biology improved with biomaterials. (A) Synthetic biology uses tools to build functionality into cells based on physiologic or synthetic inputs. This toolbox is employed via gene insertion, genetic circuits, protein circuits (adapted from Gao and Chong et al.), and/or multicellular computing (adapted from Tamsir et al.), and it enables synthesis, cell fate determination, communication, proliferation, and migration. (B) Traditional chimeric antigen receptor (CAR) T cells form YES gates that activate in response to a specific antigen. Boolean‐gated CAR T cells activate only in response to combinations of antigens, which enhances sensitivity and specificity. Common logic gates include OR (either antigen activates the CAR), AND (both antigens necessary to activate the CAR), and NIMPLY (one antigen activates the CAR, but another overrides and inhibits the activation). (C) A synthetic amphiphilic ligand acts as a cancer vaccine that homes to lymph nodes and inserts itself into the membrane of dendritic cells. An OR‐gated CAR T cell is primed with the vaccine and then exhibits potent cytotoxicity against tumour cells (adapted from Ma et al.). (D) Designer cells are encapsulated in alginate microbeads, which confer immune protection and selective permeability to prevent an inflammatory and fibrotic reaction while allowing physiologic sensing and therapeutic output (adapted from Xie et al.).

Synthetic biology re‐designs cells through genetic and physical engineering, creating a toolbox of tunable functionalities. Cells are retrofitted to sense and respond to biomarkers, enzymes, mechanical force, small molecules, or light, which allows changes in cell behavior driving behaviors such as protein synthesis, cell fate, communication, proliferation and migration (Figure 3A). Synthetic biology entered the clinic with the approval of CAR T cell therapy for relapsed and refractory leukemia and lymphoma.^74^ CAR T cells are genetically modified T cells with a synthetic receptor that senses an antigen to initiate a T cell response, and new iterations of CAR design confer Boolean logic functionality (Figure 3B). While CAR T cells represent a major victory for synthetic biology, efficacy is limited to hematologic cancers. Successful treatment of solid tumours is limited by the lack of specific antigens, poor T cell persistence in the TME and off‐tumour antigen recognition, resulting in high morbidity and poor response rates.^75^ Further, CAR T cells are prohibitively expensive to manufacture for widespread use.^76^ Thus, increased safety and efficacy and decreased cost are necessary before therapeutic immune cells can be broadly adopted against cancer. Biomaterials promise to bring these therapies closer to that ideal through enhanced manufacturing and improved performance in vivo.

Beyond CAR T cell therapy, there is a broad need for greater biocompatibility in synthetic biology‐driven cellular therapy for endocrine, autoimmune and infectious diseases. Designer cells such as insulin‐releasing cell implants may usher in a new era of diabetes treatment. However, allogeneic cells induce a robust immune response and therefore require improvements in biocompatibility for off‐the‐shelf therapies without the need for immune suppressive regimens.^35^ Engineering biomaterials provides a pathway to biocompatible and scalable designer cell implants.

### 3.1 in vivo biomaterials enhancements for T cell therapy

All cells naturally exhibit complex, logic‐gated functionality for physiologic processes such as proliferation, differentiation, and migration.^77^ For instance, T cell activation and proliferation requires primary and secondary co‐stimulation for activation and expansion.^78^ Biomaterials are used to simulate these signals with off‐the‐shelf technologies that enable widespread adoption at low cost. Several reviews thoroughly cover biomaterials for adoptive T cell therapy (ACT) and CAR T cell manufacturing ex vivo.^34^, ^79^ In the following section, we describe recent developments in biomaterial technologies for enhancing the activity of CAR T cells and ACT in vivo. We place a specific emphasis on Boolean logic‐gated CAR T cells and examples of enhancement with biomaterials.

#### 3.1.1 Biomaterials for modulation of therapeutic immune cell activity

Enhancing potency and specificity of T cell activity with biomaterials is emerging as a promising strategy against solid tumours.^74^ In an early demonstration of material implants for enhancing T cell therapeutics, Stephan et al. describe a solid polymer matrix delivering CAR T cells to a tumour resection site. The alginate matrix forms a macroporous structure that facilitates the release and expansion of T cells via a collagen mimetic peptide, enabling egress, and silica microparticles releasing an IL‐15 superagonist, promoting proliferation in vivo. The scaffolds demonstrate potent anti‐tumour efficacy in murine models of breast cancer (4T1) and ovarian cancer (ID8).^80^ Similar alginate scaffolds incorporating STING agonists eliminate heterogeneous tumours in orthotopic models of pancreatic cancer and melanoma.^81^ Finally, nitinol films and stents carrying CAR T cells molded to the shape of a resection site, providing anti‐tumour efficacy or preventing occlusion of a stented area (e.g., pancreatic duct) with tumour ingrowth.^82^

Other groups are developing CAR T cell releasing hydrogels. Hu et al. report hyaluronic acid hydrogels carrying CAR T cells, polymeric nanoparticles encapsulating IL‐15 and anti‐PDL1‐conjugated platelets. The hydrogels decrease overall tumour size in a murine model of melanoma and encourages T cell expansion in vivo.^83^ Wang et al. describe GD2‐targeted CAR T cells released from an injectable chitosan‐polyethylene glycol (PEG) hydrogel for control of retinoblastoma. The inclusion of a hydrogel delivery system limits toxicity in the retina via decreased inflammation and retinal detachment.^84^ Grosskopf et al. show that transient injectable hydrogels deliver B7H3 CAR T cells and cultivate a locally pro‐inflammatory niche via release of IL‐15 and exhibit efficacy in murine models of human medulloblastoma.^13^

Nanoparticles are a well‐established method of delivering materials to a tumour and shaping the immunomodulatory environment.^85^ iRGD‐targeted liposomes, reported by Zhang et al. deliver immunomodulatory compounds that suppress regulatory T cell (Treg) activity and stimulate invariant natural killer T cells (iNKT). This opens a therapeutic window for CAR T cells to better invade and proliferate within the TME, resulting in increased survival in models of breast cancer and glioblastoma.^86^ Tang et al. report protein nanogel particles that ‘backpack’ on T cells. The nanogels encapsulated an IL‐15 super‐agonist, which releases upon surface reduction of an activated T cell. Thus, T cells with nanogel backpacks exhibit robust proliferation in tumours and increased survival and tumour clearance in models of murine melanoma and glioblastoma.^87^

#### 3.1.2 Biomaterials for in situ generation of therapeutic T cells

Due to the expensive CAR T cell manufacturing process, in situ generation of CAR T cells or ACT is an attractive concept, allowing for off‐the‐shelf T cell therapy without allogeneic T cell transplant. ex vivo techniques for gene transfer to T cells (e.g., retroviral or lentiviral transduction) are poorly suited for in vivo use due to poor specificity, immunogenicity, and short half‐life, leading to a risk of random insertion in off‐target cells and insertional mutagenesis. Lentiviral vectors, for instance, are inactivated by human serum and lack selectivity for T cells.^88^, ^89^ While approaches for CD4 and CD8 targeted lentiviral vectors exist, such approaches involve viral vector generation and vectors specific to CD4+ and CD8+ T cells that may prove challenging to produce at scale.^90^, ^91^

Most non‐viral approaches for in situ CAR T or ACT generation utilize nanoparticles designed to home to secondary lymphoid organs such as the lymph nodes and spleen.^92^ Smith et al. describe polymeric nanocarriers specifically targeted to CD3+ T cells. The particles contained DNA encoding a CAR and a piggyback transposase for stable integration. Systemic administration of the nanoparticles demonstrates efficacy comparable to ex vivo‐generated CARs in a model of murine leukemia.^93^ The same group reports efficient delivery of mRNA to host T cells using similar nanocarriers.^94^ Rurik et al. repurposed CAR T cells to fight cardiac fibrosis using ionizable lipid NPs to generate in situ mRNA CARs by targeting CD5+ cells towards fibroblast activating protein (FAP). The FAP CARs accumulate in the spleen and travel to heart tissue to reduce fibrosis and eliminate FAP‐expressing fibroblasts.^95^

Scaffolded biomaterials also play a key role for in vivo manufacturing of CAR T cells. Agarwalla et al. utilize an alginate scaffold to produce functional CAR T cells in vivo. The scaffolds contain anti‐CD3 and anti‐CD28 monoclonal antibodies (mAbs), interleukin 2 (IL‐2) and retrovirus to activate, expand and transduce T cells. This creates functional CAR T cells with similar characteristics to ex vivo manufacturing but with improved proportion of naïve, central memory and lymphoid homing T cells. The scaffolds outperform intravenously administered CAR T cells in multiple murine models of human lymphoma.^14^

#### 3.1.3 Logic‐gated CAR T cells enhanced by biomaterials

Logic‐computation is at the forefront of next generation CAR T cell technologies. Genetic logic in CAR T cells allows for enhanced specificity and sensitivity. Common logic gates include OR,^96^ AND^97^ and NIMPLY,^98^, ^99^ with cancer antigens as inputs and cellular responses as output. However, despite the success of logic CARs in preclinical models, several limitations still exist. For example, Roybal et al. report AND‐gated CAR T cells using a synNotch gene circuit that drives CAR expression for a tumour antigen upon binding to a priming antigen. This circuit enhanced specificity and mediated killing against dual‐antigen tumours while sparing single antigen tumours (which represented healthy tissue in the model).^100^ However, Srivastava et al. later demonstrate that synNotch CAR T cells act more like IF, THEN logic gates instead of AND gates in a murine model of human lymphoma where tumour cells are intermixed with healthy tissue in the bone marrow.^101^

Using logic‐gated CARs in conjunction with biomaterials promises to overcome deficits in logic CAR T cells by conferring spatial control, bio‐orthogonal input presentation, and enhanced in vivo activation. Huang et al. report DNA scaffolded particles for tunable protein loading and synNotch CAR T cell modulation. The scaffolds present in vivo priming signals and confer spatiotemporal control. The particles allow efficient and modular loading of therapeutic or immunomodulatory proteins such as checkpoint inhibitor mAbs, IL‐2 and costimulatory ligands. This system, when used in conjunction with an AND‐gate CAR T, which contains a SynNotch CAR, induces the expression of a conventional CAR. The particles present a bio‐orthogonal protein for priming, theoretically enhancing safety by localizing CAR T cell activity. The system exhibits efficacy and spatial control in a dual‐tumour model of myelogenous leukemia.^102^

Biomaterials are also formulated into cancer vaccines that improve CAR T cell activity. Ma et al. describe a cancer vaccine that restimulates CAR T cells within the native lymph node. Designer amphiphile ligands decorated the surface of antigen presenting cells (APCs) via membrane insertion and prime CAR T cells for enhanced expansion and anti‐tumour efficacy. Systemically delivered vaccine as an albumin nanoparticle traffics to lymph nodes, and primes CAR T in vivo. The system demonstrates efficacy in immunocompetent models of mouse glioma and melanoma. They further utilized an OR‐gated CAR that targeted FITC or TRP1, a melanoma antigen. The OR logic gate allows for use of FITC or another bio‐orthogonal targeting ligand alongside an antigen of choice, where one scFv targets a tumour‐specific antigen and the other primes CAR T cells upon binding APCs (highlighted in Figure 3C).^103^

### 3.2 Designer cell therapeutics for endocrine, autoimmune and infectious diseases

While CAR T cells for cancer receive significant attention and investment due to tremendous efficacy in humans and potential in oncology, cellular therapeutics are emerging for endocrine,^104^ autoimmune,^105^ fibrotic^106^, ^107^ and infectious diseases,^108^ among others. Cellular systems offer tremendous advantages over traditional therapeutics due to sense‐and‐respond architectures afforded by genetically engineered systems, sometimes called ‘open‐loop’ genetic circuits.^109^ Open‐loop circuits confer diverse functionality to the end user, taking inputs such as electricity, shear‐stress, chemicals, heat and light.^110^, ^111^, ^112^ Other approaches utilize ‘closed‐loop’ genetic circuits which respond to natural disease biomarkers, allowing autonomous function in vivo.^15^ Both open‐ and closed‐loop systems require a prolonged expression of therapeutic gene circuits. These systems require gene circuits stably integrated into autologous cells, immune‐protected allogeneic implants or efficient in situ transfection. As with CAR T cells, autologous cells are phenotypically variable from patient to patient, and they are expensive to produce. Biomaterials promise to enhance the delivery of these cells by providing an immune‐privileged environment and enhanced, tissue‐specific gene delivery.

#### 3.2.1 Biomaterials for therapeutic cell delivery and in situ therapeutic manufacturing

Without utilizing autologous cells, lifelong immunosuppression must accompany cellular implants to prevent allorecognition and rejection long term. Islet of Langerhans transplantation from cadaveric donors results in substantial transplant mass loss and requires immune suppressive regimens.^113^ Alginate membranes for encapsulation and immune protection of implanted, allogeneic pancreatic beta cells were investigated in the 1980s.^114^ However, while transplanted cells are protected, the materials induce host immune responses, which lead to fibrosis and eventual failure of the implant.^35^, ^115^ A combinatorial screen of covalent chemical modifications yielded alginate derivatives, which elicit substantially reduced immune reactions in non‐human primates via inhibition of macrophage recognition.^116^ These alginate derivatives encapsulate functional stem‐cell derived pancreatic beta cells for at least 174 days in immunocompetent mice.^16^

An alternative approach is in situ generation of therapeutic cells. To circumvent ex vivo manufacture of therapeutic cells, some groups utilize transient in situ production of designer cells via hydrodynamic transfection of plasmid DNA.^117^, ^118^ Stable integration of DNA via nanocarriers or transient dosing with mRNA lipid nanoparticles, as demonstrated with CAR T cells, may confer cell‐type specificity and prolonged expression to these systems. Nanoparticles functionalized with cell‐specific antibodies achieve cell type specificity,^94^, ^95^ and prolonged expression can be realized via transposase integration.^93^ These technologies offer a path to in situ and autologous production of cell‐type specific, stably integrated gene circuits.

#### 3.2.2 Designer cell implants for endocrine disorders

The goal of type 1 diabetes (T1D) therapeutics is blood glucose control without patient intervention. With the availability of functional pancreatic beta cells from human pluripotent stem cells^119^ and the development of designer cells that effectively regulate insulin in vivo,^120^ strategies for integrating these cells and circuits in vivo are of paramount importance.

Closed‐loop designer cells bypass reliance on stem‐cell derived beta cells and allow fine‐tuned responses and external intervention via open‐loop safety switches.^104^ Most T1D designer cell therapies rely on alginate encapsulation methods for implantation without immune rejection, demonstrating the power of synthetic biology and biomaterials to achieve fine‐tuned physiologic responsiveness while maintaining biocompatibility. Xie et al. achieved closed‐loop insulin secretion and corrected hyperglycemia in T1D mice (highlighted in Figure 3D). They also corrected type 2 diabetes (T2D)‐related hyperglycemia via the release of glucagon‐like peptide 1. Alginate microbeads prevent immune rejection of the encapsulated cells.^120^ The Fussenegger group report similar insulin‐secreting cell implants that rely on open‐loop inputs such as oral drugs (rapamycin)^117^ and piezoelectric signaling.^121^ Additionally, designer cells have been reported that reverse diabetic ketoacidosis in mice by sensing extracellular pH and releasing insulin in response.^122^

Beyond diabetes, Rössger et al. report closed‐loop genetic circuits for treating obesity. Alginate microencapsulated cells detect fatty acid levels and produce the appetite‐suppressing peptide hormone pramlintide. Mice on high‐fat diets show significantly improved lipid levels and body weight when treated with the encapsulated designer cells.^123^ Designer cells can also regulate the control of thyroid hormones via expression of a thyroid stimulating hormone‐receptor antagonist.^124^

#### 3.2.3 Designer cell implants for autoimmunity

Autoimmunity is an emerging target of engineered cell therapy. Strategies to expand autoantigen‐specific regulatory T cells (Tregs) have generated considerable interest in adoptive cell therapy for autoimmune disease.^125^ Regulatory T cells (Tregs) are being explored for several autoimmune applications including graft‐versus‐host disease, T1D and organ transplant rejection.^126^ CARs expressed in regulatory T cells (CAR Treg) are commonly deployed in preclinical studies of collitis,^127^ transplant rejection^128^ and multiple sclerosis.^129^ Currently, no studies exist demonstrating the synergy of CAR Tregs and biomaterials. However, similar limitations apply to CAR T cells for cancer and CAR Tregs for autoimmunity, particularly if Treg isolation is imperfect and there are contaminating effector T cells in the therapeutic bolus.^130^ Biomaterials could offer similar benefits to CARs in the autoimmune space, conferring spatiotemporal control, proliferation and sensitivity to achieve efficacy with a wide margin of safety.

Designer cells offer another pathway for the treatment of autoimmune diseases through synthetic biology and biomaterials. Schukur et al. report encapsulated logic‐gated HEK293T cells to ameliorate psoriatic flares in immunocompetent BALB/c mice. The HEK293T cells quantify tumour necrosis factor and IL‐22 levels using AND‐gated synthetic signaling cascades to drive anti‐inflammatory IL‐4 and IL‐10 expression. This ‘cytokine converter’ demonstrates improved skin morphology in murine models of established psoriasis.^131^

#### 3.2.4 Designer cell implants for infectious disease

Both T cell therapies and designer cell circuits are in development for infectious diseases. For instance, anti‐HIV CARs have been studied for decades.^105^ While anti‐HIV CARs present different challenges compared to CAR T cells for cancer immunotherapy, the high cost and poor scalability of ex vivo CAR T manufacturing are shared obstacles that could be improved with biomaterials.^92^ Using alginate‐microencapsulation, Liu et al. report a closed‐loop genetic circuit in mammalian cells to sense bacteria via Toll‐like receptors and express lyostaphin to target methicillin‐resistant *Staphylococcus aureus* (MRSA), curing 100% of mice subjected to acute MRSA infections.^132^ Sedlmayer et al. describe quorum‐quenching cells to dissolve traditionally antibiotic resistant *Pseudomomas aeruginosa* biofilms.^133^

## 4 OPPORTUNITIES FOR SYNERGY IN DUALLY INTERACTIVE LIVING MATERIALS

**FIGURE 4:**
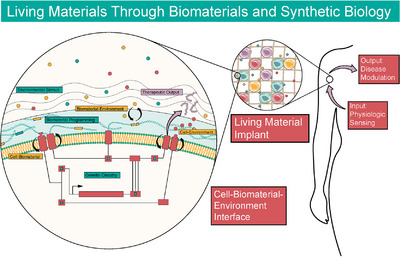
Living materials made from mammalian cell‐biomaterial hybrids. Existing technologies are converging on living material implants, which sense and respond to physiologic or pathologic stimuli and change internal properties and output based on these sensing mechanisms. Living material systems would feature response modules, which facilitate interaction between cell and biomaterial, biomaterial and environment, and environment and cell to dynamically respond and change form.

Within mammalian therapeutics, no examples of dually interactive and programmable materials and synthetic biology systems exist. All examples utilize programmable materials, which impart useful properties onto cells or vice‐versa, a one‐way interaction. Mammalian genetic circuits suffer from considerable restrictions due to the potential for toxicity, the need for impeccable pharmacokinetic control, and the difficulty in engineering biocompatible, non‐immunogenic platforms.^15^, ^112^ Developing dually interactive systems, where feedback loops and logic operations are engineered into both materials and cells, harnesses the power of programmability at the genetic and extracellular level. Imparting programmability to complex multicellular factories, which sense and respond to biological cues in vivo, allows dynamic modulation of the delivery vessel and the cellular response (Figure 4).

Living materials promise to create bidirectional interaction between cells and materials to unlock diverse functionality in synthetic systems, affording direct, real‐time control over outputs such as mechanical actuation, differentiation, ECM remodeling, in situ materials processing and many more.^134^ Work in non‐mammalian systems offers a glimpse into the potential of living biomaterials in mammalian cell‐based therapeutics. Self‐assembled biofilms made from *E. coli* genetically engineered to produce an amyloid curli nanofiber demonstrate the possibility of cell‐driven production of synthetic, composite living materials.^135^ Genetic engineering produces programmable biofilms and hydrogels that demonstrate nanoparticle templating,^136^ quorum sensing from cell‐to‐cell molecular communication,^137^ materials synthesis^138^ and patterning.^139^

Self‐assembled composite living materials combined with closed‐loop designer cells will form the basis for long sought‐after technologies such as programmable tissue regeneration, artificial endocrine tissue and eventually whole organs or organisms. We envision sophisticated therapeutics integrated into tissue and capable of modulating multiple inputs and outputs simultaneously. By leveraging the strengths of biomaterials and synthetic biology, new possibilities emerge in therapeutic systems. Advancement will require considerable development and investment in better tools in both genetic circuit design and stimuli‐responsive biomaterials. Stimuli‐responsive biomaterials produced by designer cells will be of substantial value, and logic imparted into protein systems will confer an additional layer of control.^140^ In summary the marriage of biomaterials and synthetic biology will continue to afford new living materials and functionality with potentially unprecedented performance.

The purpose of this review is to stimulate discussions, highlight recent approaches and success and provide further motivation for the development of programmable living materials and their use in medicine. The potential for programmable living materials exceeds our vision. Continued collaborative, interdisciplinary research will enable novel platforms for achieving longstanding goals in cellular therapeutics.

## CONFLICT OF INTEREST STATEMENT

E.M.B., S.A., Y.L.C., W.W. and M.W.G. are co‐inventors on a patent application, and the application is available for licensing.
